# Supplementary material for: Molecular and phylogenetic characterization of the monkeypox outbreak in the South of Spain
Source: Health Sci Rep. 2024 Mar 21;7(3):e1965. doi: 10.1002/hsr2.1965 (PMC10957719; doi:10.1002/hsr2.1965)
Supplement: Supplementary file 1 — Supporting information. [file HSR2-7-e1965-s002.docx]

**Molecular and phylogenetic characterization of the monkeypox outbreak in the South of Spain**

Supplementary Material

Contents

[Supplementary Table S1 1](#_heading=h.1fob9te)

[Supplementary Table S2 6](#_heading=h.3znysh7)

Supplementary Table S1. Monkeypox samples sequenced in this study, along with their collection date, origin and individual ENA IDs, belonging to the collective accession number PRJEB55075.

| **Sample** | **Collection date** | **Origin (Hospital and Province)** | **ENA Sample ID** |
| --- | --- | --- | --- |
| ANDmpxv00003 | 2022-06-07 | Hospital Universitario Virgen Macarena, Sevilla | SAMEA110434916 |
| ANDmpxv00005 | 2022-06-07 | Hospital Universitario Virgen del Rocio, Sevilla | SAMEA110434917 |
| ANDmpxv00007 | 2022-06-15 | Hospital Universitario Virgen del Rocio, Sevilla | SAMEA110434918 |
| ANDmpxv00008 | 2022-06-20 | Hospital Punta Europa, Algeciras (Cadiz) | SAMEA110434919 |
| ANDmpxv00010 | 2022-06-19 | Hospital Universitario Virgen del Rocio, Sevilla | SAMEA110434920 |
| ANDmpxv00011 | 2022-09-06 | Hospital Comarcal Santa Ana, Motril (Granada) | SAMEA113552868 |
| ANDmpxv00012 | 2022-06-09 | Hospital Comarcal Santa Ana, Motril (Granada) | SAMEA110434921 |
| ANDmpxv00013 | 2022-10-06 | Hospital Comarcal Santa Ana, Motril (Granada) | SAMEA113552869 |
| ANDmpxv00014 | 2022-06-13 | Hospital Universitario de Jaen, Jaen | SAMEA110434922 |
| ANDmpxv00015 | 2022-06-17 | Hospital Universitario de Jaen, Jaen | SAMEA110434923 |
| ANDmpxv00016 | 2022-06-14 | Hospital de Antequera, Antequera (Malaga) | SAMEA110434924 |
| ANDmpxv00017 | 2022-06-12 | Hospital Universitario San Cecilio, Granada | SAMEA110434925 |
| ANDmpxv00018 | 2022-06-15 | Hospital Costa del Sol, Marbella (Malaga) | SAMEA110434926 |
| ANDmpxv00019 | 2022-06-25 | Hospital Comarcal Santa Ana, Motril (Granada) | SAMEA110434927 |
| ANDmpxv00020 | 2022-06-22 | Hospital Universitario Virgen de la Victoria, Malaga | SAMEA110434928 |
| ANDmpxv00021 | 2022-06-25 | Hospital Universitario Virgen de la Victoria, Malaga | SAMEA110434929 |
| ANDmpxv00022 | 2022-06-25 | Hospital Universitario Virgen de la Victoria, Malaga | SAMEA110434930 |
| ANDmpxv00023 | 2022-06-22 | Hospital Universitario Virgen de la Victoria, Malaga | SAMEA110434931 |
| ANDmpxv00024 | 2022-06-20 | Hospital Universitario San Cecilio, Granada | SAMEA113552870 |
| ANDmpxv00025 | 2022-06-21 | Hospital Universitario San Cecilio, Granada | SAMEA110434932 |
| ANDmpxv00027 | 2022-06-17 | Hospital Costa del Sol, Marbella (Malaga) | SAMEA110434933 |
| ANDmpxv00028 | 2022-06-22 | Hospital Costa del Sol, Marbella (Malaga) | SAMEA110434934 |
| ANDmpxv00029 | 2022-06-21 | Hospital Costa del Sol, Marbella (Malaga) | SAMEA110434935 |
| ANDmpxv00030 | 2022-06-18 | Hospital QuirÃ³nsalud, Malaga | SAMEA110434936 |
| ANDmpxv00031 | 2022-06-22 | Hospital QuirÃ³nsalud, Malaga | SAMEA110434937 |
| ANDmpxv00032 | 2022-06-18 | Hospital QuirÃ³nsalud, Malaga | SAMEA110434938 |
| ANDmpxv00033 | 2022-06-21 | Hospital Universitario Reina Sofia, Cordoba | SAMEA110434939 |
| ANDmpxv00035 | 2022-06-21 | Hospital Punta Europa, Algeciras (Cadiz) | SAMEA110434940 |
| ANDmpxv00041 | 2022-06-24 | Hospital Universitario Reina Sofia, Cordoba | SAMEA110434941 |
| ANDmpxv00046 | 2022-06-20 | Hospital Universitario Virgen del Rocio, Sevilla | SAMEA110434942 |
| ANDmpxv00047 | 2022-06-27 | Hospital Universitario Virgen del Rocio, Sevilla | SAMEA113552871 |
| ANDmpxv00048 | 2022-06-26 | Hospital Universitario Virgen del Rocio, Sevilla | SAMEA110434943 |
| ANDmpxv00051 | 2022-06-26 | Hospital Universitario Virgen del Rocio, Sevilla | SAMEA110434944 |
| ANDmpxv00052 | 2022-06-29 | Hospital Universitario Reina Sofia, Cordoba | SAMEA113552872 |
| ANDmpxv00053 | 2022-06-29 | Hospital Infanta Elena, Huelva | SAMEA110434945 |
| ANDmpxv00054 | 2022-06-29 | Hospital Universitario Virgen Macarena, Sevilla | SAMEA110434946 |
| ANDmpxv00055 | 2022-06-29 | Hospital Universitario Virgen Macarena, Sevilla | SAMEA110434947 |
| ANDmpxv00056 | 2022-06-29 | Hospital Punta Europa, Algeciras (Cadiz) | SAMEA110434948 |
| ANDmpxv00058 | 2022-06-30 | Hospital de Montilla, Montilla (Cordoba) | SAMEA110434949 |
| ANDmpxv00060 | 2022-07-01 | Hospital Universitario Puerta del Mar, Cadiz | SAMEA110434950 |
| ANDmpxv00061 | 2022-06-30 | Hospital Universitario Virgen de Valme, Sevilla | SAMEA110434951 |
| ANDmpxv00064 | 2022-07-01 | Hospital Universitario Virgen del Rocio, Sevilla | SAMEA110434952 |
| ANDmpxv00066 | 2022-06-29 | Hospital La Merced, Osuna (Sevilla) | SAMEA110434953 |
| ANDmpxv00067 | 2022-07-01 | Hospital Universitario Virgen Macarena, Sevilla | SAMEA110434954 |
| ANDmpxv00068 | 2022-01-07 | Hospital Universitario Virgen Macarena, Sevilla | SAMEA113552873 |
| ANDmpxv00069 | 2022-07-03 | Hospital Universitario Virgen Macarena, Sevilla | SAMEA110434955 |
| ANDmpxv00071 | 2022-06-18 | Hospital Comarcal Santa Ana, Motril (Granada) | SAMEA110434956 |
| ANDmpxv00074 | 2022-07-01 | Hospital Universitario Virgen de la Victoria, Malaga | SAMEA110434957 |
| ANDmpxv00075 | 2022-06-22 | Hospital Universitario Virgen de la Victoria, Malaga | SAMEA110434958 |
| ANDmpxv00076 | 2022-06-22 | Hospital Universitario Virgen de la Victoria, Malaga | SAMEA110434959 |
| ANDmpxv00077 | 2022-06-22 | Hospital Universitario Virgen de la Victoria, Malaga | SAMEA110434960 |
| ANDmpxv00080 | 2022-07-06 | Hospital Universitario Virgen de la Victoria, Malaga | SAMEA110434961 |
| ANDmpxv00081 | 2022-06-22 | Hospital Universitario Virgen de la Victoria, Malaga | SAMEA110434962 |
| ANDmpxv00084 | 2022-06-19 | Hospital Universitario San Cecilio, Granada | SAMEA113552874 |
| ANDmpxv00085 | 2022-06-26 | Hospital Universitario San Cecilio, Granada | SAMEA110434963 |
| ANDmpxv00089 | 2022-06-20 | Hospital Universitario San Cecilio, Granada | SAMEA110434964 |
| ANDmpxv00090 | 2022-07-05 | Hospital Universitario San Cecilio, Granada | SAMEA110434965 |
| ANDmpxv00092 | 2022-06-20 | Hospital Universitario Virgen de la Victoria, Malaga | SAMEA110434966 |
| ANDmpxv00095 | 2022-07-06 | Hospital Universitario Reina Sofia, Cordoba | SAMEA110434967 |
| ANDmpxv00096 | 2022-07-05 | Hospital Universitario Reina Sofia, Cordoba | SAMEA110434968 |
| ANDmpxv00097 | 2022-07-05 | Hospital Universitario Virgen de Valme, Sevilla | SAMEA110434969 |
| ANDmpxv00098 | 2022-07-07 | Hospital Universitario Puerta del Mar, Cadiz | SAMEA110434970 |
| ANDmpxv00099 | 2022-07-07 | Hospital Universitario Reina Sofi­a, Cordoba | SAMEA113552875 |
| ANDmpxv00100 | 2022-07-08 | Hospital Juan Ramon Jimenez, Huelva | SAMEA110434971 |
| ANDmpxv00102 | 2022-07-08 | Hospital de Jerez, Jerez (Cadiz) | SAMEA110434972 |
| ANDmpxv00103 | 2022-07-08 | Hospital Universitario Virgen Macarena, Sevilla | SAMEA110434973 |
| ANDmpxv00105 | 2022-07-07 | Hospital de Riotinto, Riotinto (Huelva) | SAMEA110434974 |
| ANDmpxv00107 | 2022-07-07 | Hospital Universitario Virgen del Rocio, Sevilla | SAMEA110434975 |
| ANDmpxv00108 | 2022-07-08 | Hospital Universitario Virgen del Rocio, Sevilla | SAMEA110434976 |
| ANDmpxv00110 | 2022-07-10 | Hospital Universitario Virgen del Rocio, Sevilla | SAMEA110434977 |
| ANDmpxv00119 | 2022-07-14 | Hospital Infanta Elena, Huelva | SAMEA110434978 |
| ANDmpxv00121 | 2022-07-15 | Hospital Universitario Reina Sofi­a, Cordoba | SAMEA110434979 |
| ANDmpxv00123 | 2022-07-15 | Hospital Universitario Virgen Macarena, Sevilla | SAMEA110434980 |
| ANDmpxv00124 | 2022-07-12 | Hospital Universitario Puerta del Mar, Cadiz | SAMEA110434981 |
| ANDmpxv00126 | 2022-07-11 | Hospital Universitario Virgen Macarena, Sevilla | SAMEA110434982 |
| ANDmpxv00128 | 2022-07-11 | Hospital de Jerez, Jerez (Cadiz) | SAMEA110434983 |
| ANDmpxv00130 | 2022-07-12 | Hospital Universitario Virgen Macarena, Sevilla | SAMEA110434984 |
| ANDmpxv00132 | 2022-07-13 | Hospital Universitario Virgen Macarena, Sevilla | SAMEA110434985 |
| ANDmpxv00133 | 2022-07-13 | Hospital Universitario Puerta del Mar, Cadiz | SAMEA110434986 |
| ANDmpxv00135 | 2022-07-13 | Hospital Universitario Virgen del Rocio, Sevilla | SAMEA110434987 |
| ANDmpxv00136 | 2022-07-13 | Hospital Universitario Virgen del Rocio, Sevilla | SAMEA110434988 |
| ANDmpxv00137 | 2022-07-15 | Hospital Universitario Virgen del Rocio, Sevilla | SAMEA110434989 |
| ANDmpxv00138 | 2022-07-15 | Hospital Universitario Virgen del Rocio, Sevilla | SAMEA110434990 |
| ANDmpxv00139 | 2022-07-15 | Hospital Universitario Virgen del Rocio, Sevilla | SAMEA110434991 |
| ANDmpxv00140 | 2022-07-28 | Hospital Infanta Elena, Huelva | SAMEA110466390 |
| ANDmpxv00141 | 2022-07-20 | Hospital Juan Ramon Jimenez, Huelva | SAMEA110466391 |
| ANDmpxv00142 | 2022-07-20 | Hospital Universitario Puerta del Mar, Cadiz | SAMEA110466392 |
| ANDmpxv00143 | 2022-07-21 | Hospital Puerto Real, Puerto Real (Cadiz) | SAMEA110466393 |
| ANDmpxv00145 | 2022-07-26 | Hospital Infanta Margarita, Cabra (Cordoba) | SAMEA110466394 |
| ANDmpxv00146 | 2022-07-26 | Hospital Universitario Virgen de Valme, Sevilla | SAMEA110466395 |
| ANDmpxv00147 | 2022-07-26 | Hospital de La Linea de La Concepcion, Li­nea de la Concepcion (Cadiz) | SAMEA110466396 |
| ANDmpxv00148 | 2022-07-27 | Hospital Puerto Real, Puerto Real (Cadiz) | SAMEA110466397 |
| ANDmpxv00149 | 2022-07-27 | Hospital Puerto Real, Puerto Real (Cadiz) | SAMEA110466398 |
| ANDmpxv00150 | 2022-07-23 | Hospital Punta Europa, Algeciras (Cadiz) | SAMEA110466399 |
| ANDmpxv00151 | 2022-07-26 | Hospital Universitario Virgen del Rocio, Sevilla | SAMEA110466400 |
| ANDmpxv00152 | 2022-07-28 | Hospital Universitario Virgen del Rocio, Sevilla | SAMEA110466401 |
| ANDmpxv00153 | 2022-07-20 | Hospital Universitario Virgen del Rocio, Sevilla | SAMEA110466402 |
| ANDmpxv00154 | 2022-07-18 | Hospital Universitario Virgen del Rocio, Sevilla | SAMEA110466403 |
| ANDmpxv00155 | 2022-07-28 | Hospital Universitario Virgen del Rocio, Sevilla | SAMEA110466404 |
| ANDmpxv00156 | 2022-07-23 | Hospital Universitario Virgen del Rocio, Sevilla | SAMEA110466405 |
| ANDmpxv00157 | 2022-07-22 | Hospital Universitario Virgen del Rocio, Sevilla | SAMEA110466406 |
| ANDmpxv00159 | 2022-07-20 | Hospital Comarcal Santa Ana, Motril (Granada) | SAMEA110466407 |
| ANDmpxv00161 | 2022-07-13 | Hospital Universitario Torrecardenas, Almeri­a | SAMEA110466408 |
| ANDmpxv00162 | 2022-07-12 | Hospital Universitario Virgen de la Victoria, Malaga | SAMEA110466409 |
| ANDmpxv00163 | 2022-07-12 | Hospital Universitario Virgen de la Victoria, Malaga | SAMEA110466410 |
| ANDmpxv00164 | 2022-07-08 | Hospital Universitario Virgen de la Victoria, Malaga | SAMEA110466411 |
| ANDmpxv00165 | 2022-07-28 | Hospital Universitario Poniente, El Ejido (Almeria) | SAMEA110466412 |
| ANDmpxv00167 | 2022-07-21 | Hospital de Antequera, Antequera (Malaga) | SAMEA110466413 |
| ANDmpxv00169 | 2022-07-25 | Hospital Universitario San Cecilio, Granada | SAMEA110466414 |
| ANDmpxv00171 | 2022-07-27 | Hospital Universitario San Cecilio, Granada | SAMEA110466415 |
| ANDmpxv00172 | 2022-07-27 | Hospital Universitario San Cecilio, Granada | SAMEA110466416 |
| ANDmpxv00175 | 2022-07-26 | Hospital Costa del Sol, Marbella (Malaga) | SAMEA110466417 |
| ANDmpxv00177 | 2022-07-26 | Hospital Costa del Sol, Marbella (Malaga) | SAMEA110466418 |
| ANDmpxv00178 | 2022-07-26 | Hospital Costa del Sol, Marbella (Malaga) | SAMEA110466419 |
| ANDmpxv00181 | 2022-07-29 | Hospital Universitario Puerta del Mar, Cadiz | SAMEA113552876 |
| ANDmpxv00184 | 2022-08-08 | Hospital Universitario Puerta del Mar, Cadiz | SAMEA113552877 |
| ANDmpxv00188 | 2022-09-08 | Hospital Universitario Virgen Macarena, Sevilla | SAMEA113552878 |
| ANDmpxv00190 | 2022-08-08 | Hospital Universitario Virgen del Rocio, Sevilla | SAMEA113552879 |
| ANDmpxv00193 | 2022-05-08 | Hospital Universitario Virgen del Rocio, Sevilla | SAMEA113552880 |
| ANDmpxv00194 | 2022-07-29 | Hospital Universitario Virgen del Rocio | SAMEA113552881 |
| ANDmpxv00196 | 2022-09-08 | Hospital Universitario Virgen del Rocio, Sevilla | SAMEA113552882 |
| ANDmpxv00201 | 2022-02-08 | Hospital Universitario Virgen de la Victoria, Malaga | SAMEA113552883 |
| ANDmpxv00205 | 2022-03-08 | Hospital Universitario San Cecilio, Granada | SAMEA113552884 |
| ANDmpxv00207 | 2022-12-08 | Hospital Universitario San Cecilio, Granada | SAMEA113552885 |
| ANDmpxv00208 | 2022-01-08 | Hospital Universitario San Cecilio, Granada | SAMEA113552886 |
| ANDmpxv00210 | 2022-03-08 | Hospital Costa del Sol, Marbella (Malaga) | SAMEA113552887 |
| ANDmpxv00212 | 2022-08-24 | Hospital Puerto Real, Puerto Real (Cadiz) | SAMEA113552888 |
| ANDmpxv00213 | 2022-05-09 | Hospital de La Li­nea de La Concepcion, Linea de la Concepcion (Cadiz) | SAMEA113552889 |
| ANDmpxv00214 | 2022-08-17 | Hospital Puerto Real, Puerto Real (Cadiz) | SAMEA113552890 |
| ANDmpxv00215 | 2022-01-09 | Hospital Universitario Virgen del Rocio, Sevilla | SAMEA113552891 |
| ANDmpxv00216 | 2022-08-17 | Hospital Universitario Virgen del Rocio, Sevilla | SAMEA113552892 |
| ANDmpxv00217 | 2022-08-18 | Hospital Universitario Virgen del Rocio, Sevilla | SAMEA113552893 |
| ANDmpxv00218 | 2022-08-17 | Hospital Universitario Virgen del Rocio, Sevilla | SAMEA113552894 |
| ANDmpxv00219 | 2022-08-23 | Hospital Universitario Virgen del Rocio, Sevilla | SAMEA113552895 |
| ANDmpxv00220 | 2022-08-23 | Hospital Universitario Virgen del Rocio, Sevilla | SAMEA113552896 |
| ANDmpxv00222 | 2022-08-16 | Hospital de La Linea de La Concepcion, Linea de la Concepcion (Cadiz) | SAMEA113552897 |
| ANDmpxv00223 | 2022-08-XX | Hospital Universitario Virgen del Rocio, Sevilla | SAMEA113552898 |
| ANDmpxv00232 | 2022-09-21 | Hospital Universitario Virgen Macarena, Sevilla | SAMEA113552899 |
| ANDmpxv00233 | 2022-08-17 | Hospital Universitario Virgen Macarena, Sevilla | SAMEA113552900 |
| ANDmpxv00235 | 2022-08-18 | Hospital Universitario Virgen del Rocio, Sevilla | SAMEA113552901 |
| ANDmpxv00236 | 2022-08-16 | Hospital Universitario Virgen del Rocio, Sevilla | SAMEA113552902 |
| ANDmpxv00237 | 2022-08-22 | Hospital Universitario Virgen del Rocio, Sevilla | SAMEA113552903 |
| ANDmpxv00238 | 2022-09-21 | Hospital Universitario Virgen del Rocio, Sevilla | SAMEA113552904 |
| ANDmpxv00240 | 2022-09-14 | Hospital Universitario Virgen del Rocio, Sevilla | SAMEA113552905 |
| ANDmpxv00241 | 2022-08-08 | Hospital La Merced, Osuna (Sevilla) | SAMEA113552906 |
| ANDmpxv00242 | 2022-06-24 | Hospital Universitario Carlos Haya, Malaga | SAMEA113552907 |
| ANDmpxv00243 | 2022-06-14 | Hospital Universitario Carlos Haya, Malaga | SAMEA113552908 |
| ANDmpxv00244 | 2022-06-27 | Hospital Universitario Carlos Haya, Malaga | SAMEA113552909 |
| ANDmpxv00245 | 2022-06-22 | Hospital Universitario Carlos Haya, Malaga | SAMEA113552910 |
| ANDmpxv00246 | 2022-06-16 | Hospital Universitario Carlos Haya, Malaga | SAMEA113552911 |
| ANDmpxv00247 | 2022-06-17 | Hospital Universitario Carlos Haya, Malaga | SAMEA113552912 |
| ANDmpxv00248 | 2022-06-27 | Hospital Universitario Carlos Haya, Malaga | SAMEA113552913 |
| ANDmpxv00249 | 2022-01-07 | Hospital Universitario Carlos Haya, Malaga | SAMEA113552914 |
| ANDmpxv00250 | 2022-08-25 | Hospital Universitario Carlos Haya, Malaga | SAMEA113552915 |
| ANDmpxv00251 | 2022-08-07 | Hospital Universitario Carlos Haya, Malaga | SAMEA113552916 |
| ANDmpxv00252 | 2022-09-14 | Hospital Universitario Carlos Haya, Malaga | SAMEA113552917 |
| ANDmpxv00253 | 2022-11-08 | Hospital Universitario Carlos Haya, Malaga | SAMEA113552918 |
| ANDmpxv00254 | 2022-07-14 | Hospital Universitario Carlos Haya, Malaga | SAMEA113552919 |
| ANDmpxv00255 | 2022-01-08 | Hospital Universitario Carlos Haya, Malaga | SAMEA113552920 |
| ANDmpxv00256 | 2022-07-22 | Hospital Universitario Carlos Haya, Malaga | SAMEA113552921 |

Supplementary Table S2. Non-synonymous mutations found in the samples under study with respect to the reference ON563414 in NC_063383 coordinates.

| **Amino acid mutation** | **Nucleotide mutation** | **Gene** | **Uniprot ID** | **reference** | **alternative** | **Amino acid position** | **samples** | **Number of samples** |
| --- | --- | --- | --- | --- | --- | --- | --- | --- |
| NBT03_gp174:R84K | G190660A | NBT03_gp174 | A0A0F6N8V5 | R | K | 84 | ANDmpxv00001; ANDmpxv00017; ANDmpxv00046; ANDmpxv00054; ANDmpxv00055; ANDmpxv00061; ANDmpxv00069; ANDmpxv00100; ANDmpxv00105; ANDmpxv00133; ANDmpxv00137; ANDmpxv00162 | 12 |
| NBT03_gp175:Q188* | C191615T | NBT03_gp175 | Q3I9L9 | Q | * | 188 | ANDmpxv00151 | 1 |
| NBT03_gp175:R359K | G192129A | NBT03_gp175 | Q3I9L9 | R | K | 359 | ANDmpxv00058; ANDmpxv00167 | 2 |
| OPG001:S225L | C196308T | OPG001 | Q6WYZ8 | S | L | 225 | ANDmpxv00151 | 1 |
| OPG015:Q188* | G5595A | OPG015 | Q3I9L9 | Q | * | 188 | ANDmpxv00151 | 1 |
| OPG015:R359K | C5081T | OPG015 | Q3I9L9 | R | K | 359 | ANDmpxv00058; ANDmpxv00167 | 2 |
| OPG023:R311K | C11969T | OPG023 | A0A0F6N8E6 | R | K | 311 | ANDmpxv00003 | 1 |
| OPG023:S638L | G10988A | OPG023 | A0A0F6N8E6 | S | L | 638 | ANDmpxv00053 | 1 |
| OPG025:D55N | C15105T | OPG025 | A0A0F6N8R3 | D | N | 55 | ANDmpxv00020 | 1 |
| OPG025:M327I | C14287T | OPG025 | A0A0F6N8R3 | M | I | 327 | ANDmpxv00151 | 1 |
| OPG029:D15N | C17027T | OPG029 | Q3T6C8 | D | N | 15 | ANDmpxv00143 | 1 |
| OPG036:D25N | C20992T | OPG036 | A0A0F6N7F4 | D | N | 25 | ANDmpxv00010; ANDmpxv00151 | 2 |
| OPG037:D134N | C22034T | OPG037 | A0A0F6N7Y2 | D | N | 134 | ANDmpxv00056 | 1 |
| OPG042:G352E | C26043T | OPG042 | Q5IXZ5 | G | E | 352 | ANDmpxv00015 | 1 |
| OPG042:P3L | G27090A; G27091A | OPG042 | Q5IXZ5 | P | L | 3 | ANDmpxv00090 | 1 |
| OPG043:S39F | G27840A | OPG043 | Q5IXZ4 | S | F | 39 | ANDmpxv00033 | 1 |
| OPG045:D12N | C29229T | OPG045 | A0A0F6N8S5 | D | N | 12 | ANDmpxv00010 | 1 |
| OPG049:E235K | C32449T | OPG049 | A0A2L0AQQ4 | E | K | 235 | ANDmpxv00138 | 1 |
| OPG049:V296I | C32266T | OPG049 | A0A2L0AQQ4 | V | I | 296 | ANDmpxv00165 | 1 |
| OPG053:R50Q | C34542T | OPG053 | Q5IXY4 | R | Q | 50 | ANDmpxv00005 | 1 |
| OPG054:E335K | C34994T | OPG054 | A0A0F6N8X6 | E | K | 335 | ANDmpxv00097 | 1 |
| OPG055:R41C | G36963A | OPG055 | Q3I8X3 | R | C | 41 | ANDmpxv00149 | 1 |
| OPG056:E111K | C38704T | OPG056 | A0A2L0AQR3 | E | K | 111 | ANDmpxv00019 | 1 |
| OPG064:E28K | C45817T | OPG064 | Q5IXX4 | E | K | 28 | ANDmpxv00021 | 1 |
| OPG065:R28C | G46402A | OPG065 | Q5IXX3 | R | C | 28 | ANDmpxv00028 | 1 |
| OPG074:E263K | C56339T | OPG074 | A0A0F6N8J2 | E | K | 263 | ANDmpxv00123 | 1 |
| OPG074:L469F | T55719A | OPG074 | A0A0F6N8J2 | L | F | 469 | ANDmpxv00136 | 1 |
| OPG074:R665C | G55133A | OPG074 | A0A0F6N8J2 | R | C | 665 | ANDmpxv00017; ANDmpxv00046; ANDmpxv00054; ANDmpxv00055; ANDmpxv00061; ANDmpxv00069; ANDmpxv00100; ANDmpxv00105; ANDmpxv00133; ANDmpxv00137; ANDmpxv00162 | 11 |
| OPG080:D437N | C60710T | OPG080 | Q5IXW0 | D | N | 437 | ANDmpxv00096 | 1 |
| OPG094:R194H | G74360A | OPG094 | A0A0F6N8K8 | R | H | 194 | ANDmpxv00021; ANDmpxv00124; ANDmpxv00153; ANDmpxv00154 | 4 |
| OPG094:S213L | C74417T | OPG094 | A0A0F6N8K8 | S | L | 213 | ANDmpxv00019 | 1 |
| OPG102:D152N | G79538A | OPG102 | A0A0F6N8X1 | D | N | 152 | ANDmpxv00159 | 1 |
| OPG105:P178S | C81657T | OPG105 | A0A0F6N8L8 | P | S | 178 | ANDmpxv00148 | 1 |
| OPG105:S1273F | C84943T | OPG105 | A0A0F6N8L8 | S | F | 1273 | ANDmpxv00143 | 1 |
| OPG110:S92F | C89906T | OPG110 | A0A0F6N8M2 | S | F | 92 | ANDmpxv00003; ANDmpxv00020; ANDmpxv00056; ANDmpxv00095; ANDmpxv00097; ANDmpxv00121; ANDmpxv00135; ANDmpxv00143; ANDmpxv00159 | 9 |
| OPG111:D192N | G90838A | OPG111 | Q3T652 | D | N | 192 | ANDmpxv00119 | 1 |
| OPG113:K173R | A92248G | OPG113 | A0A0F6N7N0 | K | R | 173 | ANDmpxv00005 | 1 |
| OPG115:E47K | G94798A | OPG115 | Q5IXS5 | E | K | 47 | ANDmpxv00003; ANDmpxv00020; ANDmpxv00056; ANDmpxv00095; ANDmpxv00097; ANDmpxv00121; ANDmpxv00135; ANDmpxv00143; ANDmpxv00159 | 9 |
| OPG123:A249V | G104325A | OPG123 | A0A0F6N8U0 | A | V | 249 | ANDmpxv00020; ANDmpxv00056; ANDmpxv00095; ANDmpxv00121 | 4 |
| OPG125:T352I | G106600A | OPG125 | A0A0F6N8Q2 | T | I | 352 | ANDmpxv00066 | 1 |
| OPG129:D399N | C109810T | OPG129 | Q5IXR1 | D | N | 399 | ANDmpxv00149 | 1 |
| OPG129:S220F | G110346A | OPG129 | Q5IXR1 | S | F | 220 | ANDmpxv00027 | 1 |
| OPG129:S616L | G109158A | OPG129 | Q5IXR1 | S | L | 616 | ANDmpxv00163 | 1 |
| OPG136:R476Q | C118161T | OPG136 | Q5IXQ4 | R | Q | 476 | ANDmpxv00149 | 1 |
| OPG149:P145S | C127628T | OPG149 | Q5IXP1 | P | S | 145 | ANDmpxv00051 | 1 |
| OPG151:R767K | G131223A | OPG151 | Q5IXN9 | R | K | 767 | ANDmpxv00005 | 1 |
| OPG153:D440N | C136350T | OPG153 | A0A650BUA0 | D | N | 440 | ANDmpxv00135 | 1 |
| OPG153:E366K | C136572T | OPG153 | A0A650BUA0 | E | K | 366 | ANDmpxv00155 | 1 |
| OPG153:H462Y | G136284A | OPG153 | A0A650BUA0 | H | Y | 462 | ANDmpxv00097 | 1 |
| OPG163:S115F | C142545T | OPG163 | A0A0F6N9C7 | S | F | 115 | ANDmpxv00100; ANDmpxv00162 | 2 |
| OPG165:D92N | G143789A | OPG165 | Q5IXM6 | D | N | 92 | ANDmpxv00010 | 1 |
| OPG171:L61F | C147168T | OPG171 | Q5IXM3 | L | F | 61 | ANDmpxv00010 | 1 |
| OPG175:R50Q | G149590A | OPG175 | Q5IXL9 | R | Q | 50 | ANDmpxv00012 | 1 |
| OPG176:D4N | G149818A | OPG176 | A0A2L0ARH6 | D | N | 4 | ANDmpxv00028; ANDmpxv00066 | 2 |
| OPG185:E121K | G159277A | OPG185 | Q8BEJ6 | E | K | 121 | ANDmpxv00080; ANDmpxv00130; ANDmpxv00141 | 3 |
| OPG189:D54N | G163492A | OPG189 | A0A0F6NA15 | D | N | 54 | ANDmpxv00107; ANDmpxv00136 | 2 |
| OPG190:P221S | C165782T | OPG190 | Q773E2 | P | S | 221 | ANDmpxv00019 | 1 |
| OPG193:E111K | G167664A | OPG193 | A0A2L0AR39 | E | K | 111 | ANDmpxv00064; ANDmpxv00067; ANDmpxv00108; ANDmpxv00132; ANDmpxv00146 | 5 |
| OPG198:S196L | C170624T | OPG198 | A0A0F6N8W2 | S | L | 196 | ANDmpxv00119 | 1 |
| OPG205:E709K | G177780 | OPG205 | A0A0F6N8V0 | E | K | 709 | ANDmpxv00023 | 1 |
| OPG208:E269K | G180070A | OPG208 | A0A650BUW6 | E | K | 269 | ANDmpxv00102 | 1 |
| OPG209:S30* | C180606A | OPG209 | A0A0F6N9F7 | S | * | 30 | ANDmpxv00100 | 1 |
| OPG210:D1604N | G186165A | OPG210 | A0A0F6N917 | D | N | 1604 | ANDmpxv00010; ANDmpxv00014 | 2 |
| OPG210:S750L | C183604T | OPG210 | A0A0F6N917 | S | L | 750 | ANDmpxv00025 | 1 |
